# Bone Marrow Transplantation Restores Follicular Maturation and Steroid Hormones Production in a Mouse Model for Primary Ovarian Failure

**DOI:** 10.1371/journal.pone.0032462

**Published:** 2012-03-07

**Authors:** Mohsen Ghadami, Ebtehal El-Demerdash, Dong Zhang, Salama A. Salama, Awadh A. Binhazim, Anthony E. Archibong, Xinlei Chen, Billy R. Ballard, M. Ram Sairam, Ayman Al-Hendy

**Affiliations:** 1 Endocrine and Metabolism Research Institute, and Department of Medical Genetics, School of Medicine, Tehran University of Medical Sciences, Tehran, Islamic Republic of Iran; 2 Department of Obstetrics and Gynecology, Center for Women's Health Research, School of Medicine, Meharry Medical College, Nashville, Tennessee, United States of America; 3 Iranian Academic Center for Education, Culture, and Research, Tehran University of Medical Science Branch, Tehran, Islamic Republic of Iran; 4 Department of Pharmacology and Toxicology, Faculty of Pharmacy, Ain Shams University, Cairo, Egypt; 5 Department of Obstetrics and Gynecology, Baylor College of Medicine, Houston, Texas, United States of America; 6 Department of Pathology, School of Medicine, Meharry Medical College, Nashville, Tennessee, United States of America; 7 Clinical Research Institute of Montreal, Univerisité de Montréal, Montréal, Québec, Canada; National Cancer Institute, United States of America

## Abstract

Recent studies suggest that bone marrow stem cells (BMSCs) are promising grafts to treat a variety of diseases, including reproductive dysfunction. Primary ovarian failure is characterized by amenorrhea and infertility in a normal karyotype female, with an elevated serum level of follicle-stimulating hormone (FSH) and a decrease level of estrogen caused by a mutation in FSH receptor (FSHR) gene. Currently, there is no effective treatment for this condition. The phenotype of FSHR (−/−) mouse, FORKO (follitropin receptor knockout), is a suitable model to study ovarian failure in humans. Female FORKO mice have elevated FSH, decreased estrogen levels, are sterile because of the absence of folliculogenesis, and display thin uteri and small nonfunctional ovaries. In this study, we determined the effects of BMSC transplantation on reproductive physiology in this animal model. Twenty four hours post BMSC transplantation, treated animals showed detectable estroidogeneic changes in daily vaginal smear. Significant increase in total body weight and reproductive organs was observed in treated animals. Hemotoxylin and eosin (H&E) evaluation of the ovaries demonstrated significant increase in both the maturation and the total number of the follicles in treated animals. The FSH dropped to 40–50% and estrogen increased 4–5.5 times in the serum of treated animals compared to controls. The FSHR mRNA was detected in the ovaries of treated animals. Our results show that intravenously injected BMSCs were able to reach the ovaries of FORKO mice, differentiate and express FHSR gene, make FSHR responsive to FSH, resume estrogen hormone production, and restore folliculogenesis.

## Introduction

Hypergonadotropic hypogonadism accounts for up to 40% of women with primary amenorrhea [1]. Many of these cases are due to abnormalities of the sex chromosomes, such as Turner syndrome. In normal karyotype females, hypergonadotropic hypogonadism is a heterogeneous condition where the same phenotype can have different etiologies [2]. The term “resistant ovary syndrome” (ROS) is typically used to describe women with primary or secondary amenorrhea, elevated circulating endogenous FSH and luteinizing hormone (LH) levels, 46, XX karyotype, intact uterus and vagina, absence of concomitant autoimmune disease, and presence of numerous primordial follicles as evident in ovarian biopsy [3]. This condition is a common cause of primary ovarian failure [4] and is devastating for the patients who, besides being infertile, will usually face an accompanying wide range of physical and psychological problems. The possibility of achieving spontaneous pregnancy is minimal. Ovulation stimulation with human menopausal gonadotropin, as well as glucocorticoids treatment, is unsuccessful in these patients [5]. There is currently no effective treatment for this condition apart from symptomatic management with hormone replacement therapy [3]. The sole treatment option for these women is to achieve pregnancy through *in vitro* fertilization using donated eggs. The resulting pregnancy has to be supported by externally supplied hormones [3]. Histological examination of the ovaries of women with ROS reveals a normal follicular milieu [6]–[8]. The follicles, however, are arrested at the primordial stage because they cannot respond to the FSH-enhancing effect. Laboratory assessment shows high blood levels of FSH and low levels of estrogen. It has long been suspected that the main physical defect in these women is an abnormal FSH receptor that renders the ovaries insensitive or resistant to the abundant FSH in the circulation, thus the name: resistant ovary syndrome. Aittomaki *et al*. provided conclusive evidence for this concept by identifying a C to T mutation at nucleotide 566 of exon 7 of the FSHR gene located on chromosome 2p, and predicting an alanine to valine substitution at residue 189 of the FSHR protein in these patients. Measurements of cAMP suggests that this mutation greatly decreases the receptor activity and subsequently leads to ovarian failure. The disorder is heterogeneous with autosomal recessive mode of inheritance in most cases [9] .To date, variable abnormalities of pubertal development with primary or secondary amenorrhea in patients with a partial or total loss of functional mutations in different regions of the hFSHR gene have been reported [10]–[13]. Mice carrying mutated FSHR gene, the so-called FORKO (follitropin receptor knockout) mouse, is an appropriate animal model for studying human hypergonadotropic ovarian dysgenesis and infertility. Female FORKO mice display thin uteri, small ovaries, elevated serum level of FSH, decreased estrogen level, and are sterile because of the absence of folliculogenesis at the primary stages [14]. We and other investigators have shown that a variety of diseases, including reproductive dysfunction, can be treated by bone marrow stem cells (BMSCs) [15]–[17]. The existence of germline stem cells and the resultant ongoing follicle production in the ovaries of postnatal mice has been reported by Johnson, et al. 2004 [18]. Based on gene expression analyses and bone marrow transplantation (BMT) experiments using chemotherapy sterilized recipients, Johnson et *al*. [19] showed in adult female mice that a putative germline stem cells reservoir that supports oogenesis appears to reside in the bone marrow (BM). Additional transplantation studies using peripheral blood (PB) harvested from transgenic females, with germline-restricted green fluorescent protein (GFP) expression show that GFP-positive oocytes are formed in chemotherapy-treated recipient females. This suggests that putative germ cells in BM release progenitor cells into the peripheral circulation that travel to the ovaries for oocyte production [19]. Selesniemi et al. [16] showed that stem cell transplantation prevents age-related infertility in adult female mammals.

Here we show that adult bone marrow can be grafted into the ovaries of knock-out mice for FSHR gene to restore estrogen production, decrease FSH secretion, and promote folliculogenesis.

## Materials and Methods

### Animals

FSHR deficient mice (FSHR−/−) were generated as described elsewhere [14]. Animals were housed in the Meharry Medical College Animal Care Facility and provided food and water. The Institutional Animal Use and Care Committee at Meharry Medical College approved all procedures, (vide approval number 070824AAH318 on May 02, 2004 and August 07, 2007) including animal care, euthanasia, and tissue collection. FSHR-deficient (FORKO) mouse model was chosen to explore the possibility of stem cell-derived somatic cells regeneration. A FORKO breeding colony was established and all animals used in this work were generated in that colony. FSHR +/− mice were bred together and offsprings were genotyped. Mice which were FSHR +/+, +/− and −/− were obtained and used as donors and recipients in our study. In other words, all mice used in this work were syngeneic and hence we would not expect immunological reactions to the transplantation process. Six to ten weeks old female mice served as both treated and control animals.

### Screening/genotyping of mice

Mice heterozygous for FSHR (FSHR +/−) were mated, and offspring were genotyped to determine whether they were FSHR (−/−), FSHR (+/−), or WT. Briefly, DNA was extracted from 3-mm tail of tissue using the DNeasy tissue kit (QIAGEN, Valenica, CA) according to manufacturer's protocol. Extracted DNA was subjected to PCR using the following primers: 1) 5′-AAGGGACTGGCTGCTATTG-3′, 2) 5′- AGAAAAGCGGCCATTTTC-3′ set for mutated allele and 3) 5′- AGTTCAATGGCGTTCCG-3′, 4) 5′-CATGTCAGTAGTACATTAGAG-3′ set for normal allele. RED Taq Ready Mix PCR reaction containing 0.4 mM deoxynucleoside triphosphates (dNTPs), 20 mM Tris-HCL, PH 8.3, with 100 mM KCl, 3 mM MgCl2, 0.002% gelatin, stabilizers and 0.06 IU/µl *Taq* polymerase (Sigma Aldrich, St Louis, MO) was used for PCR amplification in a total volume of 50 µl of reaction containing 100 ng template DNA and 150 ng primers. PCR condition was set as a denaturation at 94°C for 5 min, followed by 30 cycles of 94°C for 45 sec, 56°C for 45 sec, 72°C for 1 min, and final extension at 72°C for 10 min. PCR products were subjected to electrophoresis in 1% agarose gel and visualized under UV light. Mice were genotyped based on the presence of only WT band (+/+), only mutated band (−/−), or both bands (+/−).

### Bone marrow transplantation of treated and control animals

Six to ten weeks old female mice were used as both treated and control animals. Female FORKO mice were given a single tail vein injection of BM collected from normal (+/+) adult syngeneic female mice. Two different control groups were used in this study: In the first group (−/−) mice were transplanted with BM cells obtained from (−/−) mice and in the second control group (+/+) mice were injected with BM cells from (−/−) mice. Bone marrow was harvested from crushed femurs and tibias of wild-type or (−/−) syngeneic female mice. In brief; animals were sacrificed; tibia and femur were clipped to small pieces with scissors after removing all muscles and connective tissues. Bone chips were thoroughly rinsed with PBS/heparin in a 50 ml tube. The washed solution containing bone marrow cells was transferred into a 50 ml tube after filtration through a 70-µm nylon mesh filter, and centrifuged at 1500 rpm for 5 min. The supernatant was aspirated and 1 ml RBC lysing buffer was added to the pellet and gently mixed for 1 min. To dilute the lysing buffer, 20 ml PBS was added and centrifuged at 250–500 g for 7 min and then the supernatant was discarded. The pellet was resuspended in complete medium. The number of cells were counted using hematocytometer and 1–2×10^7^ cells in 0.5 ml of PBS were injected into recipients intravenously via the tail vein using standard procedures. After BMT, females were mated with age-matched normal males at different time points of the experiments for possible ovulation and pregnancy during the experiments.

### Daily vaginal smear and total body weight

After IV injection of the BM, treated and control animals were weighed and tested on a daily basis for estrus cycles using vaginal smears as described previously [20]–[21].

### Tracking the donor cells

To track the donor cells, animals were sacrified from each group and organ samples were used to extract DNA. We amplified DNA from the treated and control groups by PCR, using the same primer sets and conditions used for genotyping the animals as mentioned above.

### Injection of PMSG/hCG and evaluation of oviducts for the release of oocytes and corpora lutea formation

Female mice in control and treated groups (six/group) were subjected to an ovulation induction. Briefly, folliculogenesis was induced in each mouse with an intra-peritoneal (IP) injection of 2.5 IU of pregnant mare serum gonadotropin (PMSG). Forty eight hours post PMSG, ovulation was induced in each mouse with an IP injection of 2.5 IU of human chorionic gonadotropin (hCG). Subsequently, mice were sacrificed between 10–13 hours post hCG by CO_2_ asphyxiation, following which uterus/oviduct complexes were excised and trimmed of fat. Oviducts were separated from the uterine horns with the aid of a dissecting microscope and flushed with Dulbecco, supplemented with 0.1% hyaluronidase (Sigma Chemical Co., St Louis, MO) that was preincubated at 37°C, into a watch glass. Individual oviductal flushing was examined with Nikon TMD inverted microscope at 400× magnification for the presence of ova.

### Tissue preparation and follicular growth measurement

Animals were sacrificed at 1, 3, 6, 9 and 12-week time points, after BMT, under anesthesia. Organ samples (brain, lung, heart, liver, spleen, femur, adipose, ovary, uterus, vagina and cervix) were collected, weighed and stored at −70°C until further processing. All organs were weighed by blinded lab technician who were not aware of control or treated animals. Organ weight was calculated as the percentage of total body weight. The tissues were fixed in 10% formalin overnight and embedded in paraffin. Five-micrometer-thick sections were stained with hematoxylin and eosin for light microscopic histological examination. To evaluate follicular growth, follicles were classified as preantral if they contained an oocyte with a visible nucleolus, more than one layer but less than five layers of granulosa cells, and lacked an antral space. Follicles were classified as antral if they contained an oocyte with a visible nucleolus, more than five layers of granulosa cells, and/or an antral space [22].

### Hormone assays

Blood samples were collected from treated and control animals before and after treatment. Serum FSH, luteinizing hormone (LH), estrogen and progesterone levels were measured. FSH measurements were determined by RIA using reagents provided by Dr. A.F. Parlow and the National Hormone and Peptide Program and procedures validated earlier [23]. Mouse FSH reference prep was used for assay standards and Mouse FSH antiserum (guinea pig) diluted to a final concentration of 1∶400,000 was used as primary antibody. The secondary antibody was purchased from Equitech-Bio, Inc. and was diluted to a final concentration of 1∶25. The assay has a sensitivity of 2.0 ng/ml and less than 0.5% cross-reactivity with other pituitary hormones. The intra-assay and inter-assay coefficients of variation are 6.9% and 13.3%, respectively. LH was measured in serum by a modified supersensitive two-site sandwich immunoassay [24]–[25] using monoclonal antibodies MAB1 against bovine LH and TMA (Medix Kauniainen, Finland) against the human LH-beta subunit, as described previously [24]. The tracer antibody, (kindly provided by Dr. Janet Roser, Department of Animal Science, University of California, Davis) [26] was iodinated by the chloramine T method and purified on Sephadex G-50 columns. The capture antibody was biotinylated and immobilized on avidin-coated polystyrene beads (7 mm; Nichols Institute, San Juan Capistrano, CA). Mouse LH reference preparation provided by Dr. A.F. Parlow, and the National Hormone and Peptide program was used as standard. The assay has a sensitivity of 0.07 ng/ml, and the average intra-assay and inter-assay coefficients of variation for the Quality Controls are 3.6% and 9.2%, respectively. The concentrations of estradiol-17β (E_2_) and progesterone (P_4_) were measured in sera with commercially available radioimmunoassay kits according to the manufacturer's instructions (Diagnostic Laboratories Inc., Webster, TX). The sensitivity for E_2_ assay was 4.7 pg/ml and the intra-assay coefficients of variation (CVs) were 5.3, 5.3, and 3.2% for low, medium and high E_2_ containing samples, respectively. The inter-assay CVs for E_2_ assay for the aforementioned samples were 8.1, 9.3 and 8.1%, respectively. The sensitivity for P_4_ assay was 0.1 ng/ml and the intra-assay CVs were 6.4, 3.3, 5.6% for low, medium and high P_4_-containing samples and the inter-assay CVs for the above mentioned samples containing P_4_ were 2.4, 1.7, and 3.3%, respectively.

### RNA isolation, cDNA synthesis and gene expression

Tissues were collected from treated and control animals immediately after they were sacrificed, snap-frozen, and stored at −70°C until further processing. Total RNA was extracted from ovarian and follicular tissue using the RNeasy Mini Kit (QIAGEN, Inc., Valencia, CA) according to the manufacturer's protocol. Reverse-transcriptase generation of cDNA was performed with 0.5–1 µg of total RNA using an Omniscript reverse transcriptase kit (QIAGEN) with Oligo-dT primer according to the manufacturer's protocols. Subsequent PCR analysis was carried out on 40 µl of the cDNA, and the products were analyzed by electrophoresis on a 1% agarose gel. Primer sequences for FSHR product were as follows: (F) 5′-TCTCCTTGCTGGCATTCTTG-3′ and (R) 5′-CAAGACATCATTCTGAGAGATCTCTA-3′. This primer set specifically amplified a 230 bp region of FSHR gene, which is deleted in FORKO animal models.

### Statistical analysis

Data are presented as the mean ± SEM and were analyzed by Student *t*-test with a Fischer least square difference (LSD) post hoc test using *P*≤0.05 as the level of significance.

## Results

### Daily vaginal smear

A day after IV injection of BMSCs, treated animals started to show changes in vaginal smears. They kept showing these changes throughout the experiment, however, the changes were not as regular as normal animals, i.e. longer or shorter than a normal estrus cycle. All cycles of diestrus, proestrus, estrus and metestrus were observed in treated animals, while cyclicity in control animals remained unchanged throughout the experiment.

### Donor cells tracking

**Figure 1:**
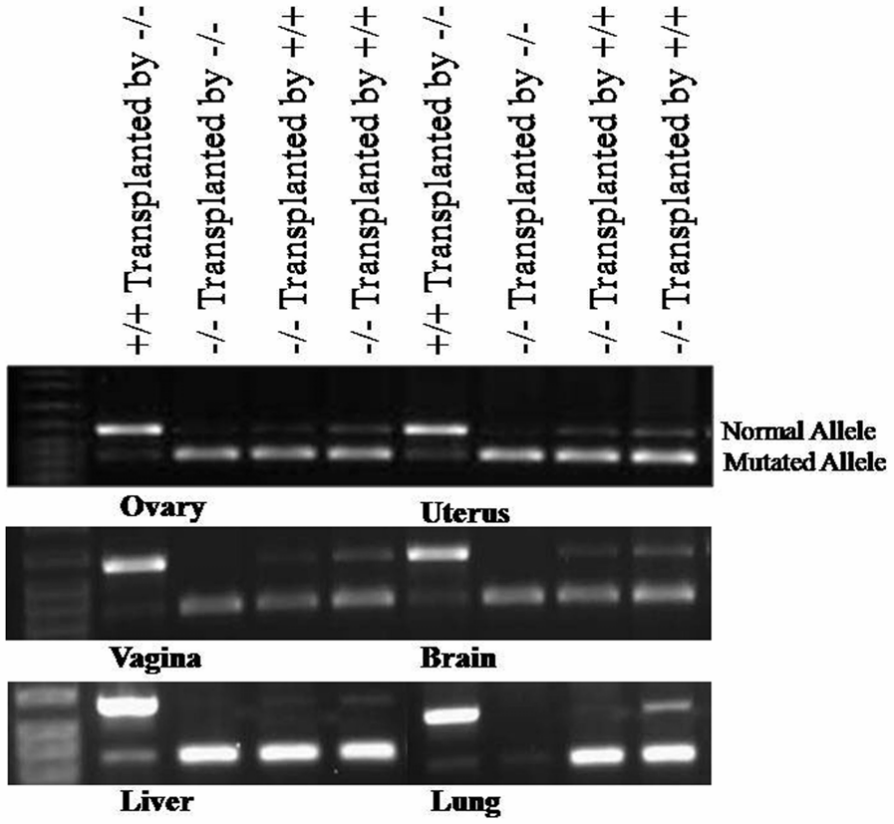
Amplified DNA from different tissues of animals transplanted by (+/+) or (−/−) donors.

Using specific primer sets for normal and mutated FSHR alleles, PCR amplification showed a normal allele band in (−/−) animals treated by BM cells from (+/+) donors in all tested organs, including brain, lung, heart, liver, spleen, ovary, uterus, vagina and femur, while the control group of (−/−) recipients injected with (−/−) donor BM did not show this normal FSHR allele in any tested organs. Mutated allele was also detected in (+/+) recipients transplanted with BM cells from (−/−) donor (Fig. 1).

### Searching for oocytes and corpora lutea formation and pregnancy

Ova were not detected in ovuductal flushing of either the control or the treated FORKO mice, indicating that the observed BMT-induced resumption in folliculogenes, based on increases in serum estrogen concentrations, was not accompanied by ovulation.

At different time points of the experiment, treated and control animals were subjected to PMSG-hCG injection and mated with age-related normal males, but no pregnancies occurred in either group within the 12-week trial period of this experiment. We also microscopically examined oviducts for possible oocyte release, but no oocytes were found in either treated or control group. The ovary histology survey showed the absence of corpora lutea in either group.

### Total body and organ weights

**Figure 2:**
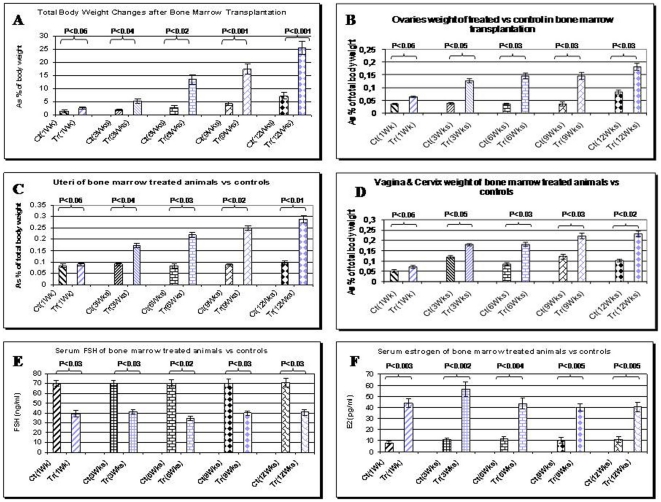
Changes of total body weight(A), ovaries(B), uterus(C), vagina and cervix(D), and serum level of FSH(E) and estrogen(F) in treated (Tr) Vs control (Ct) animals at different time points of experiment. For B, C and D organs weight considered as % of total body weight. Both treated and control group had increase in total body weight but BMT group showed significantly more increase than control group (A, P<0.03). As indicated, reproductive organs which are highly modulated by estrogen, showed remarkable increase in weight at all time points of the experiment except for the first week (for B, C, and D, a P value of less than 0.04 obtained). Bone marrow transplanted animals compare to untreated controls showed 40–50% decrease in serum FSH level (E, P<0.03) and 4–5.5 folds increase in serum estrogen (F, P<0.004) at all time points of experiment.

Significant increase in total body weight was observed in treated animals compared to control animals at different time points of treatment (P<0.03, Fig. 2A). All organs were weighed at each time point of animal sacrifice. Reproductive organs, which are highly modulated both structurally and functionally by estrogen, showed remarkable increase in weight at all time points of the experiment. Statistical analysis revealed P<0.04 for ovary, uterus, vagina and cervix for treated vs. control animals (Fig. 2B, C, and D), while changes in weight of other organs, including brain, lung, heart, liver, spleen, was not statistically significant (data not shown). We have not seen any signs of GVHD in any transplanted mice, because all animals used in this study were immunologically compatible.

### Hormonal changes

**Figure 3:**
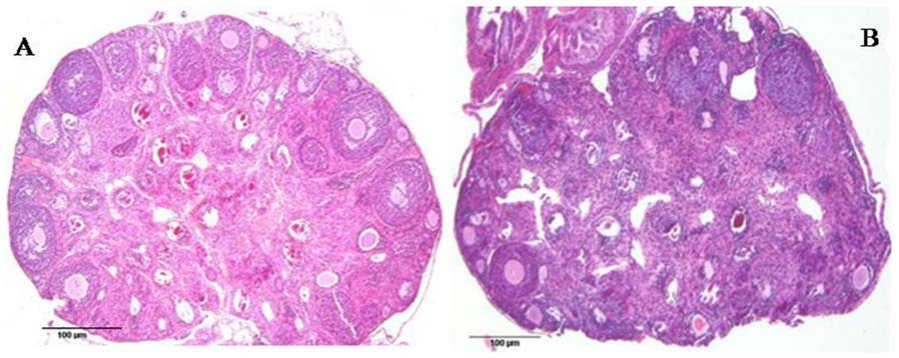
Development of the ovary in BMT animal (A) compared to untreated control animal (B). Both the total number of follicles and the number of antral follicles are significantly higher in BMT compare to control group. Histological evaluation showed on average 28±4 follicles/ovary in treated group with 8±2 follicles at the antral stage compared to only 6±2 with no follicles at antral stage in untreated control mice. Photos have been taken at the same magnification.

FORKO female mice have a high level of serum FSH, a drastic increase of at least 15-fold, compared to normal mice [14] because of the lack of the regulatory feedback at the level of FSHR. As shown in Fig. 3A, the serum FSH level decreased 40–50% in treated animals at different time points (P<0.03). In FORKO animals, estrogen is lower than normal. We observed a 4–5.5 fold increase in the level of serum estrogen in treated animals, compared to control animals (P<0.004, Fig. 3B). Changes of progesterone and LH in the serum of treated and control groups were not statistically significant (data not shown).

### Follicular growth

**Figure 4:**
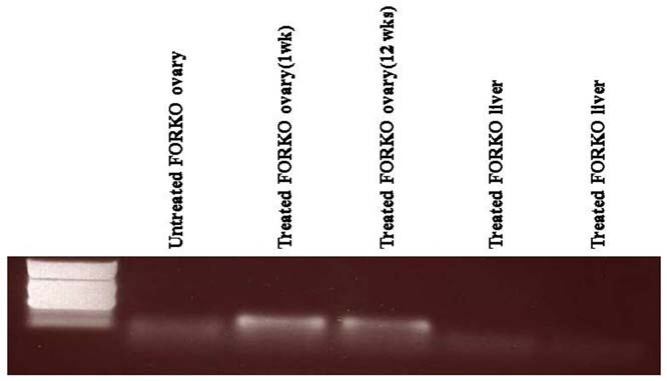
mRNA expression of FSHR gene in the ovaries of treated and control animals and in non-reproductive organs (liver) at two different time points (1 and 12 weeks). As shown, FSHR mRNA is only expressed in the ovaries of BM treated animals and there is no mRNA expression in the ovary of untreated and in non-reproductive organs of treated group.

Female FORKO mice have an overall reduced size of the ovaries and fail to develop antral follicles [27]. In our BM treated animals, both the total number of follicles and the number of antral follicles were significantly higher than control animals. On average, 28±4 follicles/ovary were observed in the BM treated group, of which 8±2 follicles were at the antral stage, while only 6±2 follicles were observed in the control group, with almost no follicles at the antral stage (Fig. 4). These data indicate that IV injection of BM cells stimulates ovarian folliculogesis to the antral stage, but not to the final ovulation. Corpus luteum was not observed in any animals. We maintained both treated and control groups with age- matched males at all steps of the experiment for possible ovulation and pregnancy, but pregnancy was not observed.

### FSHR expression

RNA extracted from the ovaries and other organs of treated and control animals showed that FSHR is exclusively expressed in the ovaries of treated animals at different time points. Expression of FSHR was not observed in other organs from the treated groups and bands were absent in the ovaries or other organs of control groups (Fig. 4). The expression of FSHR in treated animals explains the reduction of serum FSH.

## Discussion

We have shown here that cells extracted from the BM of normal syngeneic mice grafted to a preclinical FSHR knock-out mouse model (FORKO) stimulate follicular maturation to the antral stage, increase E2 production, decrease FSH secretion, and express FSHR mRNA in the ovary. Several investigators have evaluated bone marrow stem cell transplantation for the treatment of different disorders, although the mechanisms of action remain unknown and controversial. Some investigators claim that these cells differentiate, while others claim that the cells may fuse with the existing native cells, improving function of these cells by contributing their own genetic and cellular materials. Our experiments were not designed to address differentiation vs. fusion. Further experiments are clearly necessary to elucidate the exact mechanisms by which stem cells improve organ function. Our study supports the existence of stem cells in adult mammalian female's bone marrow that can influence ovarian function, even though BM transplantations in our experiment did not yield mature eggs following natural or induced ovulation. Regular ovulatory menstrual cycles come about via the coordination between the hypothalamus, pituitary, ovary, and lower reproductive tract. Normal female reproductive function requires an intact hypothalamic-pituitary unit, functional ovaries and a normally responsive uterus [28]. The murine estrous cycle generally lasts 4–5 days and can be divided into four stages called proestrus, estrus, metestrus, and diestrus. Characteristic features of specific phases, as described by Rugh et. al. [29], were as follows: a smear consisting almost exclusively of leukocytes depicted diestrus; a thin smear of equal numbers of leukocytes and elongated nucleated epithelium indicated proestrus; large cornified epithelial cells were exclusively found in estrus; and metestrus was marked by a thick smear composed of equal numbers of nucleated epithelial cells and leukocytes. Mice enter the estrus stage in the early hours of the night. Circulating levels of E2 peak prior to ovulation, which occurs at estrus, while P4 levels rise during metestrus and diestrus, and then decline from proestrus to estrus [30], [31]. Our animal model carries a mutation in FSHR gene, which lacks any response of granulosa cells to FSH. The stem cells engrafted in our experimental model made granulosa cells responsive to FSH. However, in vitro formation of granulosa cells from embryonic and skin stem cells has been reported in two different studies [32], [33]. In these studies, the authors provided evidence of estradiol biosynthesis and expression of FSHR in response to FSH in the follicles derived from skin stem cells. In a more recent study [34], the expression of FSHR in Sertoli cells by bone marrow transplantation in male mice with testicular failure has been reported. Another study has shown that premature ovarian failure and infertility observed in chemotherapy-conditioned adult female mice can be rescued by the transplantation of BM from non-treated adult female donors [17]. The functional unit within the ovary is the follicle, which consists of germ cells that become ova, and granulosa and theca cells, which produce steroid hormones. Due to the structural and functional interdependence within the follicle between the sex hormone-producing cells and the oocyte, insults that damage the endocrine cells lead to germ cell failure and infertility [28]. Mutations in the FSHR gene have been reported as a cause for primary ovarian failure (POF). Primary ovarian failure, also known as resistance ovarian syndrome (ROS), is a heterogeneous disorder characterized by amenorrhea and infertility in a normal karyotype female with an elevated serum level of follicle-stimulating hormone (FSH) and a decrease level of estrogen. We and other investigators have shown that bone marrow stem cells (BMSCs) are promising grafts for the treatment of a variety of diseases, including reproductive dysfunction [15]–[17]. The phenotype of FSHR (−/−) mouse, FORKO, is a good model to study human ROS. Female (−/−) mice have elevated serum levels of FSH, decreased E2 levels and are sterile, because of the absence of folliculogenesis at the primary follicle stage. These mice display thin uteri and small nonfunctional ovaries. The objective of our study was to determine the effects of bone marrow transplantation (BMT) on reproductive physiology of this animal model. Interestingly, the treated animals showed estroidogenic changes in the daily vaginal smears, 24 hrs after BMT, while vaginal smears in all control animals remained unchanged. Reproductive organs which are highly modulated by estrogen, both structurally and functionally, showed remarkable increase in weight at all time points of the experiment. Significant increase in both the maturation and the total number of the follicles in treated animals compared to control animals was observed. The peak follicles/ovary was observed at 3 and 6 weeks post BMT. In the study by Balla et.al. [27] regarding the dynamics of ovarian development in the FORKO mice, the authors studied ovaries of FORKO mice at different ages and concluded a postnatal and perinatal deficit consequent to FSH receptor ablation. They provided data that showed the total number of follicles was significantly lesser (*P*<0.05) in 2-day-old FORKO ovary compared to normal wild type counterpart. At 10 days of age, the total number of follicles was significantly reduced in the FORKOs compared with the wild types (*P<*0.05). These differences were spread across different developing follicle stages. They concluded that there was an age dependent change in the number of follicles in the FORKO ovary relative to the normally developing follicles in the wild type. Thus it would be conceivable that reintroducing a functioning FSHR would support an increase in size as well as number of ovarian follicles and prevent reduction the number of follicles in ovary. FSH level dropped to 40–50% and serum level of estrogen increased 4–5.5 times in treated animals compared to control animals. We detected the expression of FSHR mRNA in the ovaries of treated animals, while expression was not observed in other organs.

### Conclusion

Our results prove that intravenously injected bone marrow cells stimulate the ovaries of female FSHR (−/−) FORKO mice to express FHSR gene, restore follicular maturation and steroid hormone production. Even though treated females did not ovulate and, even after mating with age-matched males, did not conceive, the ultimate goal of the treatment of infertility, our experiment has set the stage for subsequent efforts in the treatment of this type of infertility in humans, which currently has no effective treatment. Further work is ongoing in our laboratory to better characterize this disease with the long-term goal of devising effective treatment modalities.
